# Physical Activity, Cardiorespiratory Fitness and Clustered Cardiovascular Risk in South African Primary Schoolchildren from Disadvantaged Communities: A Cross-Sectional Study

**DOI:** 10.3390/ijerph18042080

**Published:** 2021-02-21

**Authors:** Siphesihle Nqweniso, Cheryl Walter, Rosa du Randt, Larissa Adams, Johanna Beckmann, Jan Degen, Stefanie Gall, Nandi Joubert, Christin Lang, Kurt Z. Long, Ivan Müller, Madeleine Nienaber, Uwe Pühse, Harald Seelig, Danielle Smith, Peter Steinmann, Jürg Utzinger, Markus Gerber

**Affiliations:** 1 Department of Human Movement Science, Nelson Mandela University, University Way, Summerstrand, Port Elizabeth 6019, South Africa; cheryl.walter@mandela.ac.za (C.W.); rosa.durandt@mandela.ac.za (R.d.R.); larissa.adams@mandela.ac.za (L.A.); madeleine.nienaber@mandela.ac.za (M.N.); danielle.smith@mandela.ac.za (D.S.); 2 Department of Sport, Exercise and Health, University of Basel, Birsstrasse 320 B, 4052 Basel, Switzerland; johanna.beckmann@unibas.ch (J.B.); jan.degen@unibas.ch (J.D.); stefanie.gall@unibas.ch (S.G.); christin.lang@unibas.ch (C.L.); ivan.mueller@unibas.ch (I.M.); uwe.puehse@unibas.ch (U.P.); harald.seelig@unibas.ch (H.S.); markus.gerber@unibas.ch (M.G.); 3 Swiss Tropical and Public Health Institute, 4051 Basel, Switzerland; nandi.joubert@unibas.ch (N.J.); kurt.long@swisstph.ch (K.Z.L.); peter.steinmann@swisstph.ch (P.S.); juerg.utzinger@swisstph.ch (J.U.); 4 University of Basel, 4001 Basel, Switzerland

**Keywords:** blood lipids, blood pressure, body fat, cholesterol, cardiovascular risk factors, HbA1c, primary schoolchildren, physical activity, triglycerides

## Abstract

The coexistence of multiple cardiovascular risk factors has been reported in school-aged children from the age of nine years, but most evidence stems from high-income countries. This cross-sectional study aimed at describing the cardiovascular health risk, physical activity (PA) behavior and cardiorespiratory fitness (CRF) levels of South African primary schoolchildren, and at examining the associations between PA/CRF and a composite measure of cardiovascular risk. Cross-sectional data from 832 primary schoolchildren (grade 1–4) were analyzed. Total cholesterol/HDL ratio, triglycerides, systolic/diastolic blood pressure, body fat, and glycated hemoglobin were assessed as cardiovascular risk markers. Data were analyzed via mixed linear regressions and analyses of covariance. Overall, 24.2% of the participants did not meet current PA standards. Higher CRF/PA were associated with lower body fat and lower clustered cardiovascular risk (*p* < 0.05). When categorizing children into CRF/PA quartiles, a lower clustered cardiovascular risk gradient was found in children with higher CRF (*p* < 0.05) or PA (*p* < 0.05). Our data shows that higher CRF/PA is associated with lower clustered cardiovascular risk already from a young age. Given that clustered cardiovascular risk present during childhood can track into adulthood, we advocate for PA participation and a healthy weight from a young age onwards.

## 1. Introduction

Cardiovascular disease risk factors have previously been reported in adults [1] and in children and adolescents [2,3]. Clustered risk factors have been found predominantly in adults, but the coexistence of several cardiovascular risk factors is now increasingly documented in schoolchildren from the age of nine [4]. It has also been shown that clustering of cardiovascular risk factors in children is predictive of future cardiovascular disease [5]. Clustering was observed in 10–15% of children [6] and 7.1% of adolescents [7]. This is of public health concern as clustered cardiovascular risk is reported to be a stable trait from childhood to adulthood [8,9]. A review of the literature indicated that studies have consistently identified that clustering of cardiovascular risk factors were stable from childhood or adolescence to adulthood. Further studies concluded that clustering risk factors in an individual were a better and more sensitive method of assessing cardiovascular health in children [10,11]. This refined method has resulted in researchers advocating for prevention programs targeting children from a young age, as they have the potential to reduce clustered cardiovascular risk factors [8]. 

In order to develop effective intervention programs to reduce the progression of cardiovascular diseases, studies have identified factors that predict cardiovascular risk in children [12]. Physical activity (PA) and cardiorespiratory fitness (CRF), for instance, are associated with a reduction in cardiovascular risk factors [13]. Researchers have found that PA and CRF affect cardiovascular risk in different ways [14]. For instance, weight status mediates the relationship between CRF and clustered risk, while the association between PA and clustered risk is not confounded by weight status [14,15]. Further findings reveal that for children with low fitness levels, there are more chances of presenting with a clustering of cardiovascular risk compared to children with high fitness levels [2]. Ultimately, physical fitness was found to strongly predict cardiovascular disease risk compared to body weight [7]. This has major health implications, as only 50% of South African children meet the international recommendation of 60 min of PA per day [16,17]. The evidence suggests that there are differences regarding PA [18,19] and CRF levels [20] between boys and girls. Specifically, girls seem to be less active, perform relatively poorly on fitness tests and participate in more sedentary activity compared to boys [18,19]. The reasons for these gender differences might be related to the high motivation boys have to participate in PA [18], and changes during puberty such as increased testosterone production and muscle mass, while girls tend to have increased fat mass [21].

The goals of this cross-sectional study were threefold. First, to describe the cardiovascular health risk, PA behavior and CRF levels of school-aged children from disadvantaged neighborhoods in Port Elizabeth, South Africa. Second, to find out whether boys and girls, as well as younger and older children differ regarding cardiovascular health risk, PA behavior and CRF levels. Third, to examine whether independent associations exist between PA and CRF with a composite score of cardiovascular risk.

## 2. Materials and Methods

### 2.1. Study Design

This paper draws on cross-sectional baseline data of the KaziAfya study, a cluster randomized controlled trial with a 2 × 2 factorial design. The aim of the KaziAfya study was to assess the effect of a PA and multimicronutrient supplementation intervention on children’s growth, health and wellbeing in three African countries [22]. For the South African component of the study, our intention was to recruit approximately 1320 school-aged children attending grades 1–4. We randomly assigned each class per school to one of four interventions: (i) physical activity; (ii) multimicronutrient supplementation; (iii) physical activity plus multimicronutrient supplementation; and (iv) no intervention, with the latter serving as control. Baseline assessment was conducted from February to April 2019 and carried out by biokineticists, nurses and trained research assistants.

### 2.2. Participants and Procedures

Participants were recruited from four periurban primary schools (41 classes) in Port Elizabeth, South Africa. The four school were selected from two areas that are composed predominantly of Black African and Coloured children. All schools were categorized as quintile three schools. South African government schools are categorized into five quintiles, with the poorest schools classified in quintile one and the least poor in quintile five. The included schools are considered as “disadvantaged” schools as they are non-fee-paying schools. The relevant school authorities were approached before seeking contact to schools via the school principals. The principals were given detailed information about the objectives, procedures, and risks/benefits of participating in the study, they then had the opportunity to state their interest in participating in the study.

Prior to starting baseline data collection, we provided information to parents/guardians about the study. Special emphasis was placed on voluntary participation, data confidentiality, and that withdrawal from the study was allowed at any time with no negative consequences for children who wished to withdraw. Subsequently, parents/guardians were asked to provide written informed consent if they agreed for their children to take part in the study. Additionally, children provided oral assent.

Schools were included in the study if they were quintile three public schools, had facilities available to implement physical education classes and if they did not participate in another research project or clinical trial. We included children in the study if they were in grade 1–4 at baseline, their parent/guardian had signed the informed consent form, they were not participating in other research projects at the same time as our study and they did not have any adverse medical condition preventing participation in PA. Medical personnel performed a physical examination on all participants to determine if they could participate in PA. Only children with a complete data set on all cardiovascular risk markers, PA and CRF were considered for the data analyses reported in this paper.

### 2.3. Measures

To assess blood pressure, a digital blood pressure monitor (Omron® M6 AC; Hoofddorp, The Netherlands) was used. Children were seated for approximately five minutes before a measurement was taken, with a 1 min rest between each measurement. Three measurements were performed, with systolic and diastolic blood pressure measured using the mean of the last two measurements. Children’s body composition was assessed by means of bioelectrical impedance analysis (BIA) using a digital scale (Tanita MC-580; Tanita Corp., Tokyo, Japan). Using the device manufacturer’s instructions, children were asked to wear minimal clothing and stand barefoot on the metal plates of the scale, ensuring optimal contact with the plates. Cardiovascular risk markers were assessed via capillary blood sampling, with the Alere Afinion AS100 analyzer (Abbott Technologies; Abbott Park, IL, USA). A fasting blood capillary sample was taken via finger prick technique to assess total cholesterol, high density lipoprotein (HDL) cholesterol, triglycerides and glycated hemoglobin (HbA1c). The accuracy and clinical utility of the finger prick method has been described previously [23,24].

PA was assessed via a light triaxial accelerometer device (ActiGraph® wGT3X-BT; Pensacola, USA). Children wore the device consistently around the hip for seven consecutive days, except when coming into contact with water. A sample frequency of 30 Hz was used to record data. Data were analyzed using the ActiLife software version 6.13.2, with raw data set to 10 s epochs. The algorithm described by Troiano et al. [25] was used to determine nonwear time. The devices were set to collect data between 6 a.m. and midnight. Within this time frame, data was regarded valid if children wore the accelerometer for a minimum of 8 h a day, on four weekdays and one weekend day [26,27]. Specific children’s cut-points were used to determine time spent sedentary, and in light (LPA), moderate (MPA) and vigorous physical activities (VPA) [28]. An overall index for moderate-to-vigorous PA (MVPA) was generated by combining MPA and VPA.

The 20 m shuttle run test was used as the measurement of choice to assess CRF [29], with an initial speed of 8.5 km/h. We stopped the test when children failed to maintain the speed of the sound signal for two consecutive laps. A standard protocol was used to estimate maximum oxygen uptake (VO_2_max) by using the number of completed laps [30,31]. 

### 2.4. Ethical Considerations

Approval of the study was obtained from the responsible ethics committees at the Nelson Mandela University in Port Elizabeth, South Africa (reference number: H18-HEA-HMS-006) and the “Ethikkommission Nordwest- und Zentralschweiz” in Switzerland (EKNZ; reference number: Req-2018-00608). Permission was also sought from the Eastern Cape Department of Education and Department of Health, South Africa. The KaziAfya study has been registered in the ISRCTN registry (http://www.isrctn.com/ISRCTN29534081 (accessed on 9 August 2018) and was conducted in line with the study protocol [22], the principles of the Declaration of Helsinki and the guidelines of Good Clinical Practice (GCP) issued by the International Conference of Harmonisation (ICH).

### 2.5. Statistical Analysis

Normal distribution of the collected data was examined by visual inspection of normality plots and by applying the Kolmogorov–Smirnov and Shapiro–Wilk tests. Descriptive statistics are reported as mean (M) and standard deviation (SD) or number (n) and percentage (%). When severe non-normality was detected (skewness and kurtosis values of ≥|2| and ≥|7|, respectively), we applied log transformation (natural log) on the measured values before calculating inferential statistics [32]. Differences between boys and girls and between younger (5–8 years) and older children (9–13 years) were tested with univariate analyses of variance (ANOVAs). χ^2^ tests were carried out to examine whether girls/boys and younger/older children were over-/underrepresented in the group that did not meet current PA standards (≥60 min of MVPA per day). Kolmogorov–Smirnov, Shapiro–Wilk tests, descriptive statistics, ANOVAs and χ^2^ tests were carried out using SPSS Version 27 (IBM Corporation, Armonk, NY, USA) for Mac.

To examine the extent to which children’s age, sex, PA, and CRF are associated with the various cardiovascular risk markers and the composite risk measure, a series of mixed linear regression models with random intercepts for school classes were performed. Mixed linear regression models were calculated with the Mplus software (version 7, Muthen & Muthen, 1998–2020) with a robust maximum likelihood estimator (MLR). These main analyses were based on the full sample, hereby estimating missing data via full information maximum likelihood (FIML). To examine the prerequisites of FIML, Little’s missing completely at random (MCAR) test was performed, using SPSS. Findings were interpreted using the following statistical coefficients for the mixed linear regression analyses: (a) estimate (standardized Beta-weight), (b) standard error (S.E.) of the estimate, and (c) *p*-value. To determine the independent association of CRF and PA, CRF was controlled for in the analyses, when we used PA estimates to predict cardiovascular risk. By contrast, when CRF was used as a predictor, total time spent in MVPA was controlled for in the analyses. To obtain a clustered cardiovascular risk score, the individual risk factors were z-standardized and added up. In the present study, we used the following formula to calculate the clustered risk score: (systolic + diastolic blood pressure/2) + body fat + ratio of total cholesterol to HDL+triglycerides+HbA1c. This formula has been used in previous publications based on data obtained from studies carried out in Europe and the United States of America [6,33] and data from the Disease, Activity and Schoolchildren’s Health (DASH) study conducted in Port Elizabeth, South Africa [34]. To examine whether there were differences regarding the clustering of cardiovascular risk in children classified to different CRF and PA quartiles, two ANCOVAs (controlled for age and sex) were performed. The Bonferroni post hoc tests was used to analyze whether the different quartile groups differed. For all statistical analyses, we used *p* < 0.05 to denote significance. 

## 3. Results

Informed consent was obtained from 1369 children, of whom 832 children had complete data sets on the cardiovascular risk markers, PA and CRF. The descriptive statistics of all independent and dependent variables for the final sample are presented in Table 1. Kolmogorov–Smirnov and Shapiro–Wilk tests indicated that none of the dependent variables was normally distributed (*p* > 0.05). Nevertheless, severe non-normality was only observed for diastolic blood pressure and triglycerides (accordingly, these variables were log-transformed). In the mixed linear regression analyses, MLR was used to handle non-normal distribution of dependent variables. Table 1 shows that nine participants had no information with regard to their sex. However, Little’s MCAR tests showed that data were missing completely at random, χ^2^ (df = 16) = 22.7, *p* = 0.121, so FIML could be applied to impute missing data. Overall, only 24.2% of the study participants did not meet recommended PA levels, defined as <60 min MVPA per day.

Table 2 indicates that children aged 9–13 years had higher HDL cholesterol and systolic/diastolic blood pressure levels compared to younger children aged 5–8 years. Younger children reported a higher ratio of total cholesterol to HDL. Older children were more sedentary and completed more laps in the shuttle run test and had lower LPA levels and lower VO_2_max scores. Similar results were found among younger/older children not achieving recommended PA standards (χ^2^ (1832) = 1.3, *p* = 0.259, 23.0% vs. 26.6%).

Girls displayed higher values for diastolic blood pressure, body fat, total cholesterol to HDL ratio, and LPA. Additionally, girls displayed a higher clustered cardiovascular risk score compared to boys. On the other hand, boys achieved higher scores for MPA, VPA, MVPA and completed more laps in the shuttle run test. No differences were found with regard to VO_2_max. The most substantial sex-related differences were found for MVPA, with 21.9% of explained variance. Girls were less likely to meet international PA recommendations than boys (χ^2^ (1823) = 95.7, *p* = 0.000, 38.3% vs. 9.2%).

Table 3 displays information about whether CRF and PA indices reflected independent correlation with cardiovascular risk factors. After we controlled for MVPA, class-in-school, age and sex, higher CRF levels were negatively correlated with lower percentage body fat and lower clustered cardiovascular risk. A negative association was also found between estimated VO_2_max and percentage body fat, triglycerides, and clustered cardiovascular risk. Higher sedentariness levels were correlated with higher percentage body fat and higher triglycerides levels, whereas higher LPA was associated with higher systolic blood pressure. MPA was negatively correlated with body fat percentage and total cholesterol. Finally, high VPA and MVPA were both associated with lower percentage body fat and lower clustered cardiovascular risk. 

**Figure 1:**
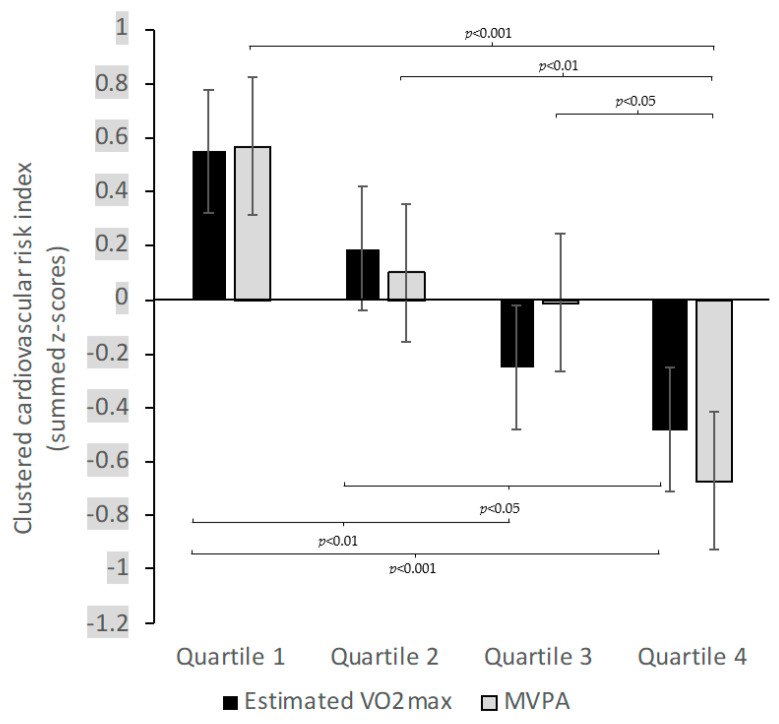
Means for clustered cardiovascular risk, stratified by quartiles of estimated VO_2_max and moderate-to-vigorous physical activity (MVPA) in schoolchildren from Port Elizabeth, South Africa, early 2019. Error bars represent standard error. Data are adjusted for age and sex.

Figure 1 illustrates that when categorizing children into quartiles based on their estimated VO_2_max or MVPA, a gradient of lower clustered cardiovascular risk between participants with higher estimated VO_2_max (F(3817) = 2.89, *p* < 0.05, η^2^ = 0.11) or MVPA (F(3817) = 2.89, *p* < 0.05, η^2^ = 0.11) appeared.

## 4. Discussion

This is one of few studies to investigate the clustering of cardiovascular risk and its association with PA and CRF in South African schoolchildren. Overall, our findings indicated that 24.2% of the study participants failed to meet the international recommendation of PA levels. We also observed that older children (9–13 years) presented with higher systolic/diastolic blood pressure, HDL levels, engaged in more sedentary activity and had lower LPA and VO_2_max scores than their younger counterparts (5–8 years). Additionally, we found similar results for children not meeting 60 min of MVPA per day in both younger (23%) and older (26.6%) children. Consistent with the existing literature [34], girls showed higher scores for most of the variables (diastolic blood pressure, percentage body fat, total cholesterol to HDL ratio and LPA) and they had a higher clustered cardiovascular risk score compared to boys. As expected, girls had a higher likelihood for not meeting the PA recommendations than boys, 38.3% vs. 9.2%, respectively. Moreover, higher CRF, VPA and MVPA were negatively associated with lower percentage body fat and lower clustered cardiovascular disease risk.

Our study found a positive result regarding PA participation, with 24.2% of the children not meeting recommended PA levels. Similar findings have been reported in children and adolescents (5–18 years) [35]. PA has been identified to have multiple beneficial health outcomes, namely cardiometabolic health, muscular fitness, bone health and CRF [36]. Among younger and older children, we found similar results regarding children not meeting the PA recommendations. Meanwhile, older children had a higher score for systolic/diastolic blood pressure and HDL cholesterol levels than younger children, who reported a higher ratio of total cholesterol to HDL. Our results corroborate those reported by Bugge and colleagues [9], who found that cardiovascular risk factors increased with age. Additionally, we found that older children were more sedentary and had lower LPA and lower VO_2_max scores. These results are significant as Andersen and colleagues [2] have demonstrated that clustered cardiovascular risk factors were not apparent in younger children (6–7 years) but started developing by age 9 in 13.8% of children, with low fitness levels being strongly related to clustering. In the longitudinal study, they were not able to explain why clustered cardiovascular disease risk was only evident after children started school, but concluded that having low fitness levels at 6–7 years predicted the development of cardiovascular risk later in life [2].

A review on children living in Sub-Saharan Africa found that girls engaged in less PA than boys, and showed higher sedentary behavior, and performed more poorly in aerobic fitness [18]. In our study, girls presented higher values for individual and clustered cardiovascular risk than boys and were less likely to meet PA recommendations. A possible reason for these findings is related to girls having higher percentage fat, which contributes to a higher prevalence of overweight/obesity, that in turn may result in individual or clustering of cardiovascular risk factors such as hypertension, dyslipidemia or impaired glucose regulation [37,38]. This contradicts the results of Musa et al. [7] who found, in Nigerian adolescents, a higher prevalence of clustered cardiovascular risk in boys than girls. Interestingly, boys in that study displayed significantly lower BMI, percentage body fat and abdominal fat, and they performed better in the fitness test than girls. According to previous cross-sectional and longitudinal surveys, overweight and unfit children have a greater risk of increasing cardiovascular disease risk than their healthy weight and fit counterparts [39,40]. Our observation regarding higher MVPA scores with regard to sex corresponds with a previous cross-sectional survey in South African school-aged children (10–15 years) that found boys to have significantly higher MVPA scores than girls [34]. However, the Healthy Lifestyle in Europe by Nutrition in Adolescence (HELENA) study found contrary results showing that adolescent girls had higher MVPA and less sedentary behavior than boys [41]. Despite the contradicting results found in the HELENA study, several studies consistently report that boys participate in more PA than girls. A recent analysis of several population-based surveys reported that there was a global trend showing girls to be less active compared to boys [42]. The study highlighted the urgency for global and national action to reduce insufficient PA, with particular emphasis on adolescent girls [42].

The results presented in this paper corroborate previous research [6,14,34], in which negative associations of higher CRF with lower percentage body fat and lower cardiovascular risk were reported. Our data showed that being sedentary was associated with higher percentage body fat and triglyceride levels, while higher LPA was associated with high systolic blood pressure. Low PA levels have been associated with increased cardiovascular risk factors. For instance, unfit and overweight children showed the highest prevalence of increased total cholesterol and systolic/diastolic blood pressure compared to their fit and normal weight counterparts [39]. In their review, Ruiz and colleagues [43] reported that low fitness levels increased the likelihood of cardiovascular disease risk 5.7 times in boys and 3.6 times in girls. We found a significant negative correlation between VPA and MVPA and percentage body fat and clustered cardiovascular risk. Previous cross-sectional studies reported that clustered cardiovascular risk could be lowered by at least 0.06-SD with every 1-SD increase in VPA [44], whereas a 0.5-SD increase in MVPA was associated with approximately 30% reduction in clustered risk [45]. Furthermore, lower clustered cardiovascular risk among children with higher CRF and MVPA levels was observed. Similar to our findings, a longitudinal study found that, compared to children in the most fit quartile, those in the lowest fitness quartile had a 34.9 times greater risk of having clustered cardiovascular risk [2].

The strengths of our study were that we used measures of PA and cardiovascular risk markers from a relatively large sample of schoolchildren from grades 1–4. We used validated and objective methods to measure PA and CRF (accelerometry and 20 m shuttle run test). Additionally, we made use of a summed score of risk markers related to cardiovascular disease. This clustered risk score is relatively stable and can compensate for the fluctuations in individual risk factors [6]. For data analyses, we used statistical software that can compensate for missing data in a meaningful way. Likewise, we used a robust estimator to deal with non-normally distributed data. 

Despite the strengths of our study, the findings should be interpreted with caution as we observed several limitations. We were unable to report inference about causality and its direction due to the cross-sectional nature of the study. We did not find any differences based on children’s ethnic background (Black African and Coloured), and therefore decided not to include this variable as a potential confounder. We also did not assess whether children engaged in intense PA the day before data assessment. This is something that was not done in previous studies investigating the relationship between PA and CRF [6,14]. Furthermore, our findings cannot be generalized to the entire South African population as children from one province were included in the investigation. Finally, we cannot generalize our findings to children from different social strata attending more wealthy schools or rural schools. Although several confounding factors were controlled for, it is conceivable that other factors such as genetic variations, dietary patterns and energy intake might have influenced our findings.

## 5. Conclusions

Our findings add to the body of knowledge showing a rise in both individual and clustered cardiovascular risk in children. Most children in our sample engaged in sufficient PA, which has positive consequences due to the beneficial effect of PA on cardiovascular health. Moreover, we were able to corroborate that PA and CRF are associated with clustered cardiovascular risk, respectively. Given the accumulating evidence that clustered cardiovascular risk tracks from childhood into adulthood, more focus should be directed at promoting PA and a healthy weight from a young age onwards.

## Figures and Tables

**Table 1:** Descriptive statistics of all study variables, for the total sample (*N* = 832).

**Predictor variables**	***M***	***SD***	**Min**	**Max**	**Skew**	**Kurt**
Sedentary activity (min/day)	610.8	68.4	339.8	852.3	−0.2	0.5
Light physical activity (min/day)	324.5	43.2	157.6	461.9	−0.2	0.1
Moderate physical activity (min/day)	56.5	16.9	12.2	122.6	0.4	−0.1
Vigorous physical activity (min/day)	25.0	12.9	3.4	115.5	1.5	4.5
Moderate-to-vigorous physical activity (min/day)	81.4	28.2	15.7	212.0	0.7	0.8
Completed laps in 20 m shuttle run test	21.9	13.3	3	113	1.9	5.9
Estimated VO_2_max (mL/kg/min)	47.6	3.8	37.9	65.0	0.7	1.7
Outcome variables	*M*	*SD*	Min	Max	Skew	Kurt
Systolic blood pressure (mm Hg)	101.7	11.9	67.5	177.0	0.6	2.0
Diastolic blood pressure (mm Hg) ^a^	63.5	9.6	40.5	141.5	1.5 (0.5)	7.3 (1.8)
Body fat (%)	22.7	5.3	9.3	48.8	1.3	2.8
Total cholesterol (mmol/L)	3.7	0.6	2.6	6.3	0.4	0.2
HDL cholesterol (mmol/L)	1.3	0.3	0.5	2.6	0.7	1.2
Total cholesterol: HDL cholesterol ratio	3.1	0.7	1.6	6.5	0.8	1.3
Triglycerides (mmol/L) ^a^	0.8	0.3	0.5	3.1	2.8 (0.9)	16.1 (1.4)
HbA1c (%)	5.4	0.3	3.2	6.4	−0.9	6.7
Clustered risk score	0.0	2.5	−6.8	12.1	0.7	1.1
Potential confounders (metric)	*M*	*SD*	Min	Max	Skew	Kurt
Age (years)	8.3	1.4	5.7	13.2	0.3	−0.7
Potential confounders (nominal)	*N*	%				
Sex ^b^						
Girls	420	51.0				
Boys	403	49.0				

Notes. M = Mean; N = Number; SD = Standard deviation; Skew = Skewness; Kurt = Kurtosis; HDL = High density lipoprotein; HbA1c = glycated hemoglobin ^a^ Scores for diastolic blood pressure and triglycerides were log-transformed (skewness and kurtosis values presented in brackets). ^b^ 9 children with missing values for sex.

**Table 2:** Differences in outcome and predictor variables, based on children’s age and sex.

Age	5–8 Years		9–13 Years			
	***M***	***SD***	***M***	***SD***	***F***	**η^2^**
Systolic blood pressure (mm Hg)	100.5	12.0	104.1	11.4	16.9 ***	0.020
Diastolic blood pressure (mm Hg) ^a^	62.8	9.6	64.8	9.4	8.8 **	0.011
Body fat (%)	22.8	5.0	22.3	5.9	1.7	0.002
Total cholesterol (mmol/L)	3.6	0.6	3.7	0.6	2.1	0.003
HDL cholesterol (mmol/L)	1.2	0.3	1.3	0.3	5.8 *	0.007
Total cholesterol: HDL cholesterol ratio	3.1	0.7	3.0	0.6	5.6 *	0.007
Triglycerides (mmol/L) ^a^	0.7	0.2	0.8	0.2	2.6	0.003
HbA1c (%)	5.4	0.2	5.4	0.3	1.8	0.002
Clustered risk score	−0.1	2.4	0.1	2.6	1.1	0.001
Sedentary activity (min/day)	604.8	67.8	623.3	68.0	13.6 ***	0.016
Light physical activity (min/day)	328.6	42.7	315.0	43.1	16.0 ***	0.019
Moderate physical activity (min/day)	56.5	16.6	56.3	17.7	0.0	0.000
Vigorous physical activity (min/day)	25.2	13.0	24.5	12.7	0.4	0.001
Moderate-to-vigorous physical activity (min/day)	81.7	28.2	80.8	28.3	0.2	0.000
Completed laps in 20 m shuttle run test	20.1	11.1	25.7	16.2	34.4 ***	0.040
Estimated VO_2_max (mL/kg/min)	48.6	3.1	45.6	4.4	128.8 ***	0.134
Sex ^b^	Girls		Boys			
Systolic blood pressure (mm Hg)	101.4	12.1	102.2	11.7	1.1	0.001
Diastolic blood pressure (mm Hg) ^a^	64.3	9.7	62.7	9.5	6.1 *	0.007
Body fat (%)	24.5	5.1	20.7	4.8	118.6 ***	0.126
Total cholesterol (mmol/L)	3.7	0.6	3.6	0.6	0.7	0.001
HDL cholesterol (mmol/L)	1.2	0.3	1.3	0.3	3.3	0.004
Total cholesterol: HDL cholesterol ratio	3.1	0.7	3.0	0.7	5.8 *	0.007
Triglycerides (mmol/L) ^a^	0.8	0.3	0.7	0.2	2.2	0.003
HbA1c (%)	5.4	0.2	5.4	0.3	0.2	0.000
Clustered risk score	0.5	2.5	−0.5	2.4	33.8 ***	0.040
Sedentary activity	629.0	63.0	592.1	67.9	65.2 ***	0.074
Light physical activity (min/day)	318.3	43.7	331.0	41.9	18.1 ***	0.022
Moderate physical activity (min/day)	48.8	13.9	64.5	16.1	224.0 ***	0.214
Vigorous physical activity (min/day)	19.7	9.1	30.4	13.9	171.2 ***	0.173
Moderate-to-vigorous physical activity (min/day)	68.5	21.6	94.9	28.0	230.7 ***	0.219
Completed laps in 20 m shuttle run test	20.3	10.7	23.7	15.4	13.1 ***	0.016
Estimated VO_2_max (mL/kg/min)	47.5	3.5	47.8	4.1	1.6	0.002

Notes: HDL = High density lipoprotein; HbA1c = glycated hemoglobin. Age: 5–8 years (*n* = 561, 67.4%), 9–13 years (*n =* 271, 32.6%); Sex: boys (*n* = 403, 49.0%), girls (*n* = 420, 51.0%). ^a^ *F* and η^2^ values calculated with log-transformed values. ^b^ 9 children with missing values for sex. * *p* < 0.05. ** *p* < 0.01. *** *p* < 0.001.

**Table 3:** Prediction of cardiovascular health risk markers with physical activity and cardiorespiratory fitness among South African schoolchildren.

	Laps Completed in 20 m Shuttle Run		Estimated VO_2_max (mL/kg/min)		Sedentary (min/day)		LPA (min/day)		MPA (min/day)		VPA (min/day)		MVPA (min/day)	
	Controlled for CRF/Accelerometer Wear Time or MVPA, and Potential Confounders (Age, Sex) ^a,b^													
	Estimate	S.E.	Estimate	S.E.	Estimate	S.E.	Estimate	S.E.	Estimate	S.E.	Estimate	S.E.	Estimate	S.E.
Systolic blood pressure (mm Hg)	−0.02	0.04	−0.02	0.04	−0.09	0.05	0.10 **	0.04	0.01	0.04	0.00	0.03	0.00	0.04
Diastolic blood pressure (mm Hg) ^c^	−0.03	0.03	−0.04	0.03	0.02	0.04	0.03	0.04	−0.07	0.04	−0.04	0.04	−0.04	0.04
Body fat (%)	−0.19 ***	0.04	−0.18 ***	0.04	0.09 *	0.04	0.02	0.03	−0.16 ***	0.04	−0.24 ***	0.03	−0.22 ***	0.03
Total cholesterol (mmol/L)	−0.02	0.04	0.06	0.04	0.07	0.04	−0.04	0.03	−0.08 *	0.04	−0.04	0.04	−0.07	0.04
HDL cholesterol (mmol/L)	−0.01	0.03	0.00	0.04	0.02	0.04	−0.01	0.04	−0.04	0.03	0.00	0.04	0.00	0.04
Total cholesterol: HDL cholesterol ratio	0.02	0.03	0.04	0.03	0.03	0.04	−0.03	0.04	0.00	0.04	0.00	0.04	0.00	0.04
Triglycerides (mmol/L) ^c^	−0.05	0.03	−0.10 **	0.04	0.10 **	0.04	−0.04	0.03	0.01	0.03	0.05	0.03	0.03	0.04
HbA1c (%)	0.00	0.04	0.01	0.03	−0.01	0.05	−0.01	0.04	0.04	0.04	0.01	0.04	0.00	0.04
Clustered risk score	−0.12 ***	0.03	−0.11 **	0.03	0.04	0.04	0.00	0.03	−0.05	0.03	−0.09 *	0.03	−0.07 *	0.03

Notes: CRF = Cardiorespiratory fitness; LPA = Light physical activity; MPA = Moderate physical activity; VPA = Vigorous physical activity; MVPA = Moderate-to-vigorous physical activity; HDL = High density lipoprotein; HbA1c = glycated hemoglobin ^a^ If laps completed or estimated VO_2_max are used as predictors, the regression analyses are controlled for MVPA and potential confounders (age, sex). If physical activity indicators are the predictor, the analyses are controlled for VO_2_max, total accelerometer devices wear time and potential confounders (age, sex). ^b^ Due to the clustered nature of the data, class-in-school was considered as a random intercept across all analyses. ^c^ Log-transformed values are used as outcome variable. * *p* < 0.05. ** *p* < 0.01. *** *p* < 0.001.

## Data Availability

All data analyzed during this study will be included in the published articles.
